# Research on Vibration Reduction Performance of Electromagnetic Active Seat Suspension Based on Sliding Mode Control

**DOI:** 10.3390/s22155916

**Published:** 2022-08-08

**Authors:** Pengshu Xie, Yusong Che, Zhengbin Liu, Guoqiang Wang

**Affiliations:** School of Mechanical and Aerospace Engineering, Jilin University, Changchun 130025, China

**Keywords:** active seat suspension, electromagnetic levitation, integral sliding mode control, state feedback control

## Abstract

Vehicle seats have a significant impact on the comfort of passengers. The development of seats is a field in which scholars are widely concerned. In this study, we add an electromagnetic levitation structure and design a new active seat suspension based on the passive seat suspension. Then, simulation research is carried out based on a C-level road surface combined with integral sliding mode control and state feedback control. The results show that both state feedback control and integral sliding mode control positively affect vehicle seat vibration reduction, and integral sliding mode control has a better anti-interference effect than state feedback control. At the same time, it is proved that the seat suspension has good working characteristics and economy.

## 1. Introduction

The vibration reduction system of a car is mainly composed of three parts: tires, body suspension, and seat suspension. The car seat is an important part of the vehicle and the last link in the vibration attenuation of the car. Changing the stiffness damping of tires and the body suspension will affect the driving performance of the car, while changing the seat suspension has little effect on the handling and drivability of the car. In addition, the manufacturing cost of the seat is low, and the manufacturing cycle is short, so improving the shock-filtering performance of the seat is a low-cost and high-efficiency method, which is of great significance for improving the riding comfort of the driver and passengers.

Low-frequency and large-amplitude vibrations cause various hazards to the human body, including bone pain, low back pain, cardiovascular disease, gastrointestinal disease, and increased risk of cancer [1,2]. In addition, large-amplitude vibrations cause great harm to the human muscle system and spine [3]. According to the experimental results, 4–10 Hz is the sensitive range of the human body [4]. In this range, resonance will occur in some areas of the body, and the body will be greatly endangered.

At present, the most common seat vibration reduction method is passive vibration reduction, that is, vibration reduction through passive suspension seats. The passive suspension seat has a simple structure, low cost, and convenient maintenance. However, since the stiffness damping of the passive suspension seat is fixed, the vibration reduction effect when dealing with complex road surfaces is unsatisfactory. To adapt to more complex road surfaces and achieve a more comfortable driving experience, active vibration reduction control methods have been proposed. An active vibration reduction control system obtains the running state of the suspension through a sensor, calculates and outputs the corresponding active control force through an algorithm, and performs vibration reduction control on the seat in real time.

**Figure 1:**
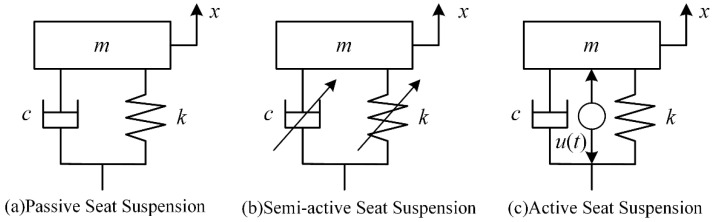
Schematic diagram of types of seat suspension.

The seat has the longest contact with the driver and is an important part of the vehicle’s vibration damping system. The seat suspension consists of springs, dampers, and other structures that improve the ride comfort by damping vibrations transmitted from the body. According to the suspension structure, seat suspensions can be divided into passive suspension, semi-active suspension, and active suspension [5]. The three types of seat suspension are shown in Figure 1. The stiffness and the coefficient for passive seat suspension are not variables. The semi-active seat suspension collects information about the suspension and vehicle body through sensors and can adjust the stiffness and damping values in real time, so that the system can attenuate the vibration in real time. An active seat suspension adds actuators to a passive seat suspension to generate control forces to suppress vibration, which consumes energy.

At present, mainstream seat optimization mainly focuses on two aspects: the optimization of the structure and research into, and optimization of, control methods. Oshinoya et al. [6] designed an active suspension system for a small vehicle to improve the riding comfort of the driver’s seat using the optimal control method, and the effectiveness of the system was verified by experiments. Maciejewski et al. [7] described the simulated dynamic response of an active vibration isolation pneumatic seat and developed and analyzed a three-feedback-loop control system based on the described active vibration isolation system. Sun et al. [8] designed a dynamic output feedback controller with an order equal to the plant, according to the actual situation of the active seat suspension system, and transformed the controller design into a convex optimization problem by using the effective multiplier expansion. This was verified by an example with specific and random road disturbances. Pan and Hao [9] proposed a five-DOF vehicle analysis mathematical model with an active seat air spring suspension system to improve the driver’s comfort. The results showed that the active seat suspension could reduce the vertical vibration acceleration of the driver more effectively than a passive seat suspension. Maciejewski [10] proposed a design method for an active vibration damping control system for the seat suspension, studied a seat with a pneumatic suspension, and shaped its vibration isolation characteristics by an appropriate selection of controller settings. Pan et al. [11] proposed a seven-DOF half-car dynamic model including a cab mounting system and seat suspension system to study the performance of the active seat suspension. The results showed that the active seat suspension could significantly improve the performance of automotive seats. Maciejewski et al. [12] studied the robustness of the proposed control system with respect to different load qualities, through computer simulation and experimental research. Gan et al. [13] proposed an active seat system to reduce the vibration levels transmitted to the seat pan and the bodies of passengers under low-frequency periodic excitation. The experimental results showed that the system had good robustness and stability.

Ning et al. [14] designed two actuators for the seat suspension, each of which had one rotating motor and one gear reducer, and verified the feasibility of the suspension system through tests. Ning et al. [15] designed a static output feedback $H_{\infty }$ controller with friction compensation to reduce the vibration of the seat. Maciejewski et al. [16] discussed a horizontal seat suspension using pneumatic muscles for active vibration control. Chouinard et al. [17] proposed a simple and economical active seat suspension using a controlled slippage magnetorheological (MR) actuator. The test results showed that the performance of the active seat suspension could match the performance of commercial alternatives. Alfadhli et al. [18] proposed the control of an active seat with vehicle suspension preview information. Ning et al. [19] proposed an integrated active and semi-active seat suspension for heavy duty vehicles and established its prototype. An integrated control algorithm using measurable variables was designed for the proposed seat prototype. Maciejewski et al. [20] introduced the control design of an active horizontal seat suspension with an electromagnetic actuator and verified the effectiveness of the active suspension system through experiments. Zhao et al. [21] established a five-DOF driver and seat suspension system model for active vibration control and developed a full-state feedback controller to reduce the human vibration in the seat suspension system. Xia et al. [22] proposed a hybrid controller using an advanced electromagnetic damper (EMD) system to meet the requirements of vibration isolation and energy saving for seat suspensions. Cvok et al. [23] introduced the design of a rig that used a linear electric servomotor to exert accurately controlled vertical vibrations on the driver’s seat, which could provide a basis for extending ride comfort evaluation research in different directions.

Zhang et al. [24] proposed an adaptive fuzzy fault-tolerant control for a seat active suspension system with an actuator fault. They proved the effectiveness of this method through simulation. Yang et al. [25] proposed a discrete nine-DOF driver’s seat active suspension model. The L2 feedback algorithm was used to solve the optimal feedback matrix of the model, and the adaptive Kalman filter algorithm was used to replace the linear Kalman filter. The results showed that the new algorithm and model significantly improved the driver comfort. Zhang et al. [26] studied the effect of a delayed resonator on the vibration reduction performance of a vehicle active seat suspension. The results showed that the delayed resonator could greatly suppress the seat vibration response regardless of the road’s simple harmonic excitation or random excitation. Maciejewski et al. [27] discussed an innovative active control for a horizontal seat suspension system in the context of realistic input vibrations that occurred in the cabins of agricultural tractors. Laboratory measurements on the seated human body have shown improved comfort of drivers under fore-and-aft vibrations. Liu et al. [28] proposed an event-triggered tracking control for active seat suspension systems with time-varying full-state constraints. The feasibility and the rationality of this method were proved by the simulation analysis of a real example of a seat suspension system.

Vehicle seats have a significant impact on the comfort of passengers, and therefore it is necessary to study active seat suspension systems. The main contribution of this paper is to propose a new active seat suspension structure that is characterized by adding an electromagnetic levitation structure to the passive seat. The function of the electromagnetic levitation structure is to provide active control forces for the active seat system. By establishing a “seat–body–tire” three-degrees-of-freedom dynamic model that incorporates an electromagnetic force, the model is feedback linearized. The electromagnet current is changed by methods such as integral sliding mode control, to control the vibration displacement and acceleration of the seat. The results show that the integral sliding mode control method has excellent anti-interference ability. The electromagnetic active seat suspension based on integral sliding mode control can effectively attenuate the vibration of the seat.

The remainder of this paper is organized as follows: Section 2 of this paper establishes the dynamic model of the seat; Section 3 discusses the design of the controller based on integral sliding mode control and state feedback control; Section 4 discusses the simulation experiments; Section 5 presents the simulation results and the discussion; and Section 6 draws the conclusions.

## 2. Dynamic Model

### 2.1. Establishment of “Seat–Body–Tire” Dynamic Model

**Figure 2:**
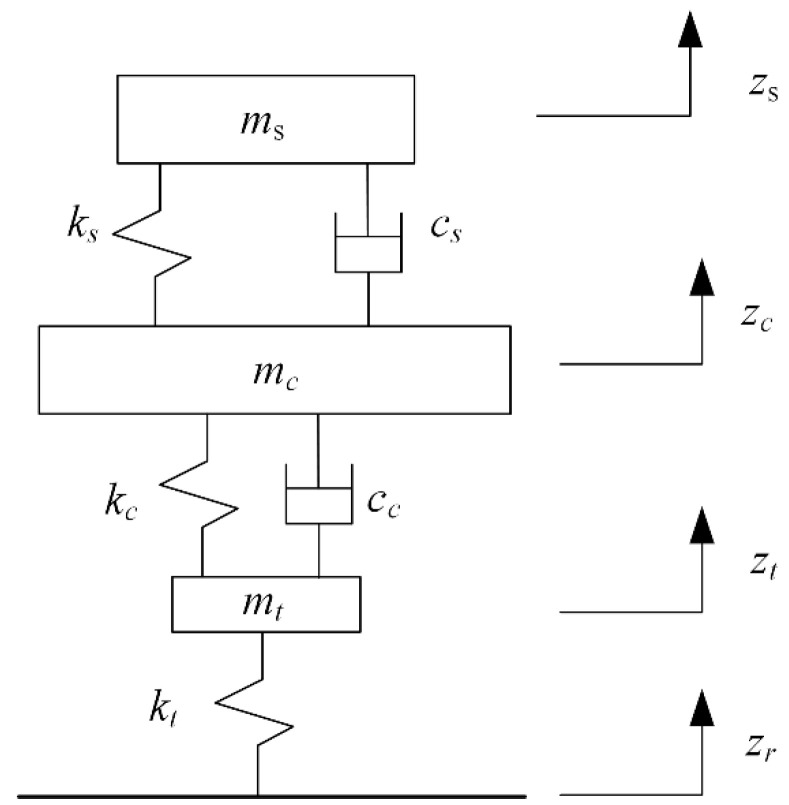
Three-degrees-of-freedom dynamic model of “seat–body–tire” system.

The three-degrees-of-freedom dynamic model for a quarter-vehicle model is shown in Figure 2. The “seat–body–tire” system of the vehicle is simplified into a three-DOF model composed of the mass block, spring, and damping element. The model can directly reflect the information on seat acceleration, seat displacement, body acceleration, suspension dynamic deformation, and tire dynamic load. In addition, because the model involves fewer suspension structure parameters, the amount of calculation required for simulation and control is small.

According to Figure 2, the motion equation of the following quarter-vehicle model is established as follows:

(1) $$\left\{\begin{array}{l} m_{s}{\ddot{z}}_{s}=k_{s}\left(z_{c}-z_{s}\right)+c_{s}\left({\dot{z}}_{c}-{\dot{z}}_{s}\right)\\ m_{c}{\ddot{z}}_{c}=k_{c}\left(z_{t}-z_{c}\right)+c_{c}\left({\dot{z}}_{t}-{\dot{z}}_{c}\right)-k_{s}\left(z_{c}-z_{s}\right)-c_{s}\left({\dot{z}}_{c}-{\dot{z}}_{s}\right)\\ m_{t}{\ddot{z}}_{t}=k_{t}\left(z_{r}-z_{t}\right)-k_{c}\left(z_{t}-z_{c}\right)-c_{c}\left({\dot{z}}_{t}-{\dot{z}}_{c}\right) \end{array}\right.$$

where $m_{s}$ is the mass of the seat and the person, $k_{s}$ is the seat suspension stiffness, $c_{s}$ is the damping for the seat suspension, $m_{c}$ is the body mass, $k_{c}$ is the body suspension stiffness, $c_{c}$ is the damping for the body suspension, $m_{t}$ is the mass of the tire, and $k_{t}$ is the stiffness of the tire. In addition, $z_{s}$, $z_{c}$, $z_{t}$, and $z_{r}$ are the seat displacement, body displacement, tire displacement, and road excitation, respectively.

According to the above dynamic equations, the dynamic simulation module of the seat passive suspension system was built in Simulink. At the same time, road excitation with a speed of 60 km/h and road level C was taken as the input of the model. Seat vertical displacement and seat vertical acceleration were selected as the main evaluation indexes of the seat suspension, to evaluate the ride comfort of vehicle drivers.

### 2.2. Electromagnetic Vibration Damping System

#### 2.2.1. Structure of Electromagnetic Vibration Reduction System

**Figure 3:**
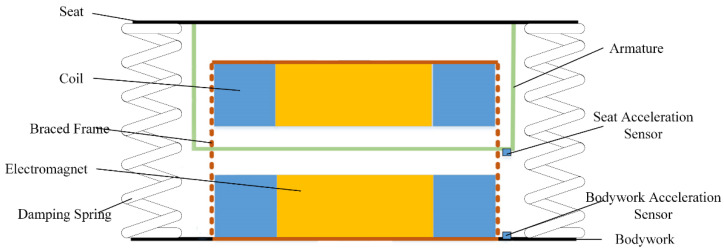
Structure of electromagnetic vibration reduction system.

The electromagnetic vibration damping structure designed in this study is shown in Figure 3. The vibration reduction system is mainly composed of four parts: the electromagnet, seat armature, controller, and actuator. The actuator includes an electromagnet and a corresponding power amplifier. When the electromagnet winding is energized, an electromagnetic attraction force is generated on the armature. When vibration occurs, one of the electromagnets is energized and the other electromagnet is de-energized. In this way, a change in the direction of the electromagnetic attraction force can be quickly realized. As long as the current in the electromagnet winding is controlled, displacement control of the seat can be realized. However, this balance between the electromagnetic force and gravity is unstable, because the magnitude of the electromagnetic force between the electromagnet and the floating body is inversely proportional to the square of their distance, that is, the smaller the distance, the greater the force and the greater the distance, the smaller the force. Therefore, if this balance is disturbed by a tiny amount, it will be destroyed. Hence, closed-loop control of the entire system must be established. The sensor picks up the displacement signal of the seat online, and the controller processes the acceleration, speed, displacement of the seat, and vibration displacement signal of the body accordingly and generates a control signal. The power amplifier generates the required control current according to the control signal, and sends it to the electromagnet coil, thereby generating a magnetic force in the executive electromagnet. According to the corresponding changes in the acceleration of the seat and the car body, the magnitude of the electromagnetic force is changed, so that the vibration of the seat can reach the desired control value.

#### 2.2.2. Dynamic Model of Electromagnetic Vibration Damping System

**Figure 4:**
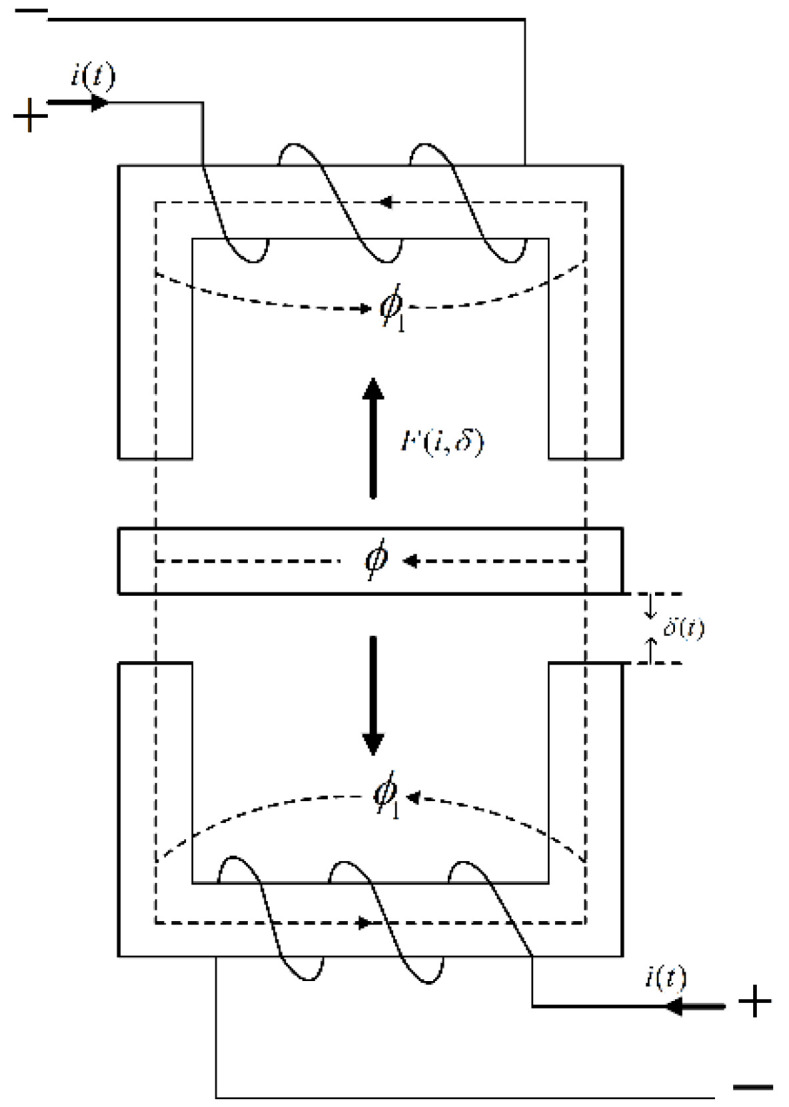
Schematic diagram of the structure of the electromagnetic vibration reduction system.

A structural schematic diagram of the electromagnetic vibration damping system is shown in Figure 4. The upper and lower ends are electromagnets, and the middle is an armature connected to the seat. Here, i(t) is the control current of the electromagnet, ϕ is the air gap flux, $\phi_{1}$ is the leakage flux, and δ(t) represents the gap between the armature and the magnet. Furthermore, F(i,δ) is the electromagnetic force.

It is assumed that the magnetic flux leakage can be ignored and the resistance in the electromagnet and armature can be neglected. It is also considered that the magnetic potential drops evenly in the air gap and the electromagnet has no movement in the horizontal direction.

(1) Establishment of electromagnetic force equation:
  (1) Idealized assumptions: (a) the armature is rigid and the stiffness coefficient is large enough; (b) the armature mass is evenly distributed; (c) when the armature is in the balanced position, the air gap between the armature and the upper and lower electromagnets is the same and is very small, to ensure that the magnetic line of force passes through vertically; (d) the magnetic flux passes through the magnetic circuit with the cross section S of the magnetic pole; (e)the permeability is $\mu_{c}\gg 1$; and (f) flux leakage is ignored.
  (2) The electromagnetic force equation is expressed by the coil current i and the air gap δ.

The magnetic reluctance of the magnetic circuit has the following relationship:

(2) $$R=\frac{2\delta }{\mu_{0}S}$$

The magnetic flux density of the air gap between the armature and the electromagnet can be written as:

(3) $$B=\frac{\phi }{S\cos\varphi }$$

where ϕ is the air gap flux and φ is the included angle between the magnetic line of force and the vertical line on the magnet surface. According to hypothesis (d), when the air gap is very small, it can be considered that the magnetic line of force passes vertically through the magnetic pole, that is, φ=0.

According to

(4) Ni=Rϕ

the electromagnetic force of the system is

(5) $$F=\frac{N^{2}i^{2}\mu_{0}S}{4\delta^{2}}$$

where $\mu_{0}$ is the vacuum permeability, $\mu_{0}=4\pi \times 10^{-7}\;H/m$. In addition, S is the conductive area of the electromagnet, N is the number of turns of the coil, δ is the distance between the electromagnet and the armature, i is the control current in the excitation coil, and F is the electromagnetic force generated by the electromagnet.

**Figure 5:**
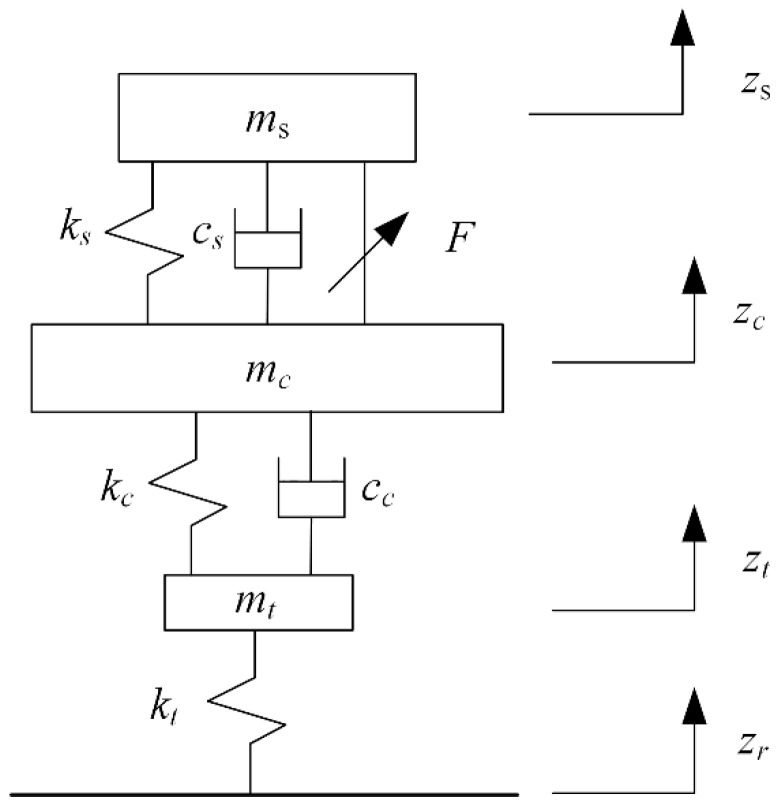
Three-degrees-of-freedom model of “seat–body–tire” system with electromagnetic force.

(2) Establishment of the three-degrees-of-freedom model and dynamic equation of “seat–body–tire” system:

Based on the model established in Figure 2, the electromagnetic force is introduced to establish the “seat–body–tire” model, as shown in Figure 5.

It is assumed that the disturbances of uncertain factors such as a sudden voltage change are ignored, that is, the seat is only subject to the excitation electromagnetic force F(i,δ) and the spring force and damping force given to it by the suspension. Carrying out a force analysis on the seat, the dynamic equation in the vertical direction of the seat can be expressed as:

(6) $$\left\{\begin{array}{l} m_{s}{\ddot{z}}_{s}=k_{s}\left(z_{c}-z_{s}\right)+c_{s}\left({\dot{z}}_{c}-{\dot{z}}_{s}\right)-F\left(i,\delta \right)\\ m_{c}{\ddot{z}}_{c}=k_{c}\left(z_{t}-z_{c}\right)+c_{c}\left({\dot{z}}_{t}-{\dot{z}}_{c}\right)-k_{s}\left(z_{c}-z_{s}\right)-c_{s}\left({\dot{z}}_{c}-{\dot{z}}_{s}\right)\\ m_{t}{\ddot{z}}_{t}=k_{t}\left(z_{r}-z_{t}\right)-k_{c}\left(z_{t}-z_{c}\right)-c_{c}\left({\dot{z}}_{t}-{\dot{z}}_{c}\right) \end{array}\right.+F\left(i,\delta \right)$$

The calculation formula of *K* is as follows:

(7) $$K=\frac{\mu_{0}N^{2}S}{4}$$

The purpose of this paper is to control the vibration displacement of the seat as a fixed percentage of the vibration displacement of the vehicle body. Therefore, substituting Equations (5) and (7) into (6), we obtain:

(8) $$m_{s}{\ddot{z}}_{s}=k_{s}z_{c}+c_{s}{\dot{z}}_{c}-k_{s}z_{s}-c_{s}{\dot{z}}_{s}-K\frac{i^{2}}{\delta^{2}}$$

The stroke of the armature is:

(9) $$z\left(t\right)=z_{s}-z_{c}$$

The initial position of the armature is $z_{0}$, and hence the gap between the upper magnet and the armature is

(10) $$\delta_{u}=z_{0}+z_{c}-z_{s}$$

The magnet clearance is preliminarily set to 0.4 m, so the clearance of the lower magnet is:

(11) $$\delta_{d}=0.4-\left(z_{0}+z_{c}-z_{s}\right)$$

When $F\left(i,\delta \right)>0$, the direction of the electromagnetic force on the vibration reduction platform is downward and $\delta =\delta_{d}$. When $F\left(i,\delta \right)<0$, the direction of the electromagnetic force on the vibration damping platform is upward and $\delta =\delta_{u}$. Since the value $z_{c}$ can be measured in real time, δ becomes a function with variables $z_{s}$ and $z_{c}$, i.e., $\delta =\delta \left(z_{s}\right)$.

### 2.3. Establishment of State-Space Equation and Feedback Linearization

#### 2.3.1. Electromagnetic Vibration Damping Seat System Model

We select the status variable $x_{1}\left(t\right)=z_{s}$, $x_{2}\left(t\right)={\dot{x}}_{1}\left(t\right)$, where $\delta \left(x_{1}\right)$ is a primary function of $x_{1}$, making the control quantity $u=i^{2}$. Then, we have

(12) $$x={\left[\begin{array}{cc} x_{1} & x_{2} \end{array}\right]}^{T}={\left[\begin{array}{cc} z_{s} & {\dot{z}}_{s} \end{array}\right]}^{T}$$

From the equation of motion (Equation (8)):

(13) $${\ddot{z}}_{s}=\frac{k_{s}z_{c}+c_{s}{\dot{z}}_{c}-k_{s}x_{1}-c_{s}x_{2}-K\frac{i^{2}}{\delta {\left(x_{1}\right)}^{2}}}{m}$$

where in order to simplify the expression, m is used to replace $m_{s}$.

From the above, the nonlinear state-space equation of the electromagnetic vibration reduction system can be obtained as follows:

(14) $$\left\{\begin{array}{l} {\dot{x}}_{1}=x_{2}\\ {\dot{x}}_{2}=\frac{k_{s}z_{c}+c_{s}{\dot{z}}_{c}-k_{s}x_{1}-c_{s}x_{2}-K\frac{i^{2}}{\delta {\left(x_{1}\right)}^{2}}}{m} \end{array}\right.$$

The equation of state can be reduced to:

(15) $$\left\{\begin{array}{l} \dot{x}=f\left(x\right)+g\left(x\right)u\\ y=h\left(x\right) \end{array}\right.$$

where

(16) $$\left\{\begin{array}{l} f\left(x\right)=\left[\begin{array}{c} x_{2}\\ \frac{k_{s}z_{c}+c_{s}{\dot{z}}_{c}-k_{s}x_{1}-c_{s}x_{2}}{m} \end{array}\right]\\ g\left(x\right)=\left[\begin{array}{c} 0\\ \frac{-K}{\delta {\left(x_{1}\right)}^{2}m} \end{array}\right]\\ u\left(t\right)=i^{2}\\ h\left(x\right)=x_{1} \end{array}\right.$$

**Figure 6:**
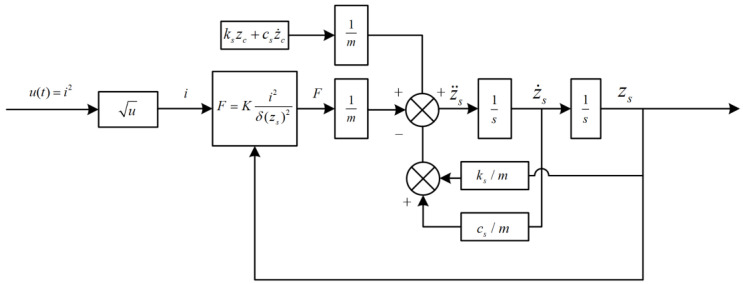
Open-loop structure of second-order system.

This model takes the current as input, and the open-loop structure of the system is shown in Figure 6.

#### 2.3.2. Linearization Process

(1) Test relative order condition

We find the Lie derivatives of Equation (16) and obtain:

(17) $$\left\{\begin{array}{l} L_{g}h\left(x\right)=\frac{\partial h}{\partial x}g\left(x\right)=\left[\begin{array}{cc} 1 & 0 \end{array}\right]\left[\begin{array}{c} 0\\ \frac{-K}{\delta {\left(x_{1}\right)}^{2}m} \end{array}\right]=0\\ L_{f}h\left(x\right)=\frac{\partial h}{\partial x}f\left(x\right)=\left[\begin{array}{cc} 1 & 0 \end{array}\right]\left[\begin{array}{c} x_{2}\\ \frac{k_{s}z_{c}+c_{s}{\dot{z}}_{c}-k_{s}x_{1}-c_{s}x_{2}}{m} \end{array}\right]=x_{2}\\ L_{g}L_{f}h\left(x\right)=\frac{\partial L_{f}h\left(x\right)}{\partial x}g\left(x\right)=\left[\begin{array}{cc} 0 & 1 \end{array}\right]\left[\begin{array}{c} 0\\ \frac{-K}{\delta {\left(x_{1}\right)}^{2}m} \end{array}\right]=\frac{-K}{\delta {\left(x_{1}\right)}^{2}m} \end{array}\right.$$

When $x_{1}\neq \infty$, the relative order is γ=2, which is equal to the order of the system and meets the condition.

(2) Linearization calculation:

The selected feedback rule is:

(18) $$u=\alpha \left(x\right)+\beta \left(x\right)v$$

where $\alpha \left(x\right)=-\frac{L_{f}^{2}h\left(x\right)}{L_{g}L_{f}^{}h\left(x\right)}=\frac{\delta {\left(x_{1}\right)}^{2}\left(k_{s}z_{c}+c_{s}{\dot{z}}_{c}-k_{s}x_{1}-c_{s}x_{2}\right)}{K}$, $\beta \left(x\right)=\frac{1}{L_{g}L_{f}^{}h\left(x\right)}=\frac{-\delta {\left(x_{1}\right)}^{2}m}{K}$.

We set the linearized state variable as:

(19) $$z={\left[\begin{array}{cc} z_{1} & z_{2} \end{array}\right]}^{T}$$

The state changes to:

(20) $$z=\phi \left(x\right)=\left[\begin{array}{c} h\left(x\right)\\ L_{f}h\left(x\right) \end{array}\right]=\left[\begin{array}{c} x_{1}\\ x_{2} \end{array}\right]$$

Then, we have:

(21) $$z={\left[\begin{array}{cc} z_{1} & z_{2} \end{array}\right]}^{T}={\left[\begin{array}{cc} x_{1} & x_{2} \end{array}\right]}^{T}={\left[\begin{array}{cc} z_{s} & {\dot{z}}_{s} \end{array}\right]}^{T}$$

From [29]:

(22) $$\left\{\begin{array}{l} Az=\frac{\partial \phi \left(x\right)}{\partial x}\left(f\left(x\right)+g\left(x\right)\alpha \left(x\right)\right)=\left[\begin{array}{cc} 0 & 1\\ 0 & 0 \end{array}\right]\left[\begin{array}{c} x_{1}\\ x_{2} \end{array}\right]\\ B=\frac{\partial \phi \left(x\right)}{\partial x}\left(g\left(x\right)\beta \left(x\right)\right)=\left[\begin{array}{cc} 1 & 0\\ 0 & 1 \end{array}\right]\left\{\left[\begin{array}{c} 0\\ \frac{-K}{\delta {\left(x_{1}\right)}^{2}m} \end{array}\right]\cdot \frac{\delta {\left(x_{1}\right)}^{2}m}{K}\right\}=\left[\begin{array}{c} 0\\ 1 \end{array}\right] \end{array}\right.$$

and $rank\left[\begin{array}{cc} B & AB \end{array}\right]=rank\left[\begin{array}{cc} 0 & 1\\ 1 & 0 \end{array}\right]=2$, i.e., the rank of the matrix is the order of the system (order 2), and the system is controllable. Therefore, the linear model of the electromagnetic vibration damping system after linearization is:

(23) $$\left\{\begin{array}{l} \dot{z}=\left[\begin{array}{cc} 0 & 1\\ 0 & 0 \end{array}\right]z+\left[\begin{array}{c} 0\\ 1 \end{array}\right]v\\ y=\left[\begin{array}{cc} 1 & 0 \end{array}\right]z \end{array}\right.$$

where

(24) $$v=\frac{u-\alpha }{\beta }=\frac{k_{s}z_{c}+c_{s}{\dot{z}}_{c}-k_{s}x_{1}-c_{s}{\dot{x}}_{1}}{m}-\frac{Ki^{2}}{\delta {\left(x_{1}\right)}^{2}m}$$

## 3. Design of Feedback Controller

### 3.1. Design of State Feedback Controller

The linearized state-space equation of the system is as follows:

(25) $$\left\{\begin{array}{l} \dot{z}=\left[\begin{array}{cc} 0 & 1\\ 0 & 0 \end{array}\right]z+\left[\begin{array}{c} 0\\ 1 \end{array}\right]v\\ y=\left[\begin{array}{cc} 1 & 0 \end{array}\right]z \end{array}\right.$$

Since $A=\left[\begin{array}{cc} 0 & 1\\ 0 & 0 \end{array}\right]$, $B=\left[\begin{array}{c} 0\\ 1 \end{array}\right]$, $C=\left[\begin{array}{cc} 1 & 0 \end{array}\right]$, the system controllability matrix is $M=\left[\begin{array}{cc} B & AB \end{array}\right]=\left[\begin{array}{cc} 0 & 1\\ 1 & 0 \end{array}\right]$, and the observable matrix is $N=\left[\begin{array}{c} C\\ CA \end{array}\right]=\left[\begin{array}{cc} 1 & 0\\ 0 & 1 \end{array}\right]$. Since Rank(M)=Rank(N)=2, the system can be both observed and controlled, and poles can be arbitrarily assigned.

The design feedback control law is as follows:

(26) $$v=Lv_{c}-K_{T}z$$

The dynamic indexes of the transformation system meet the following conditions: (1) output overshoot σ≤3%; (2) peak time $t_{p}\leq 0.15s$; and (3) static error $e_{p}=0$, where, $K_{T}=\left[\begin{array}{cc} k_{1} & k_{2} \end{array}\right]$, $K_{T}$ is the state feedback gain matrix, $v_{c}$ is the error between the theoretical value and the actual value, and R is the input transformation coefficient.

The current control law can be obtained according to Equation (24):

(27) $$i=\delta \left(x_{1}\right)\sqrt{m\left(\frac{k_{s}z_{c}+c_{s}{\dot{z}}_{c}-k_{s}x_{1}-c_{s}{\dot{x}}_{1}}{m}-v\right)/K}$$

According to:

(28) $$\left\{\begin{array}{l} \sigma =e^{-\xi \pi /\sqrt{1-\xi^{2}}}\leq 3\%\\ t_{p}=\pi /\left(\omega_{n}\sqrt{1-\xi^{2}}\right)\leq 0.15\;s \end{array}\right.$$

we can obtain:

(29) $$\left\{\begin{array}{l} \xi \geq 0.74\\ \omega_{n}\geq 31.39 \end{array}\right.$$

and then take ξ=0.75, $\omega_{n}=40$. The required poles are:

(30) $$s=-\xi \omega_{n}\pm j\omega_{n}\sqrt{1-\xi^{2}}=-30\pm 17.5j$$

Since

(31) $$\left(s+30-j17.5\right)\left(s+30+j17.5\right)=s^{2}+60s+1206.25$$

we have $k_{1}=1206.25$, $k_{2}=60$.

The closed-loop transfer function after introducing feedback is:

(32) $$G\left(s\right)=\frac{L}{s^{2}+60s+1206.25}$$

We need the static error of the system $e_{p}=0$, in order to make the static amplification factor equal to 1. According to the final value theorem:

(33) $$\underset{s\to 0}{\lim\;}G\left(s\right)=\underset{s\to 0}{\lim}\frac{L}{s^{2}+60s+1206.25}=1$$

where L=1206.25. In summary, we can obtain:

(34) $$v=Lv_{c}-K_{T}z=1206.25v_{c}-\left[\begin{array}{cc} 1206.25 & 60 \end{array}\right]\left[\begin{array}{c} z_{1}\\ z_{2} \end{array}\right]=1206.25\left(v_{c}-z_{1}\right)-60z_{2}$$

**Figure 7:**
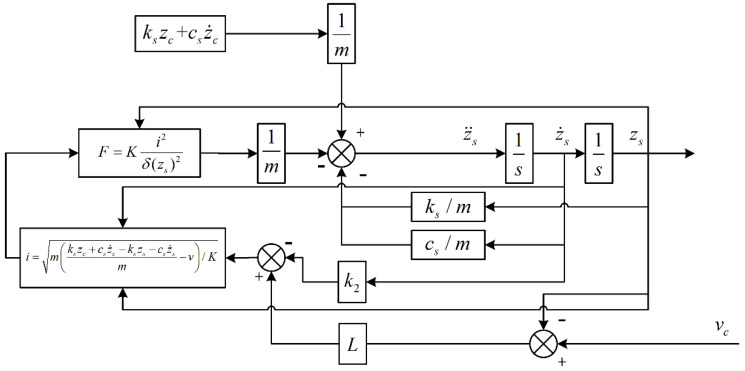
Structure block diagram of state feedback control system.

The corresponding state feedback control system was built according to the established equation, and its structure is shown in Figure 7.

### 3.2. Design of Integral Sliding Mode Controller

The displacement of the actual output of the electromagnetic vibration damping system seat is $z_{s}$, and the expected output seat displacement is $y_{d}$. The displacement position error is defined as e, where

(35) $$e=z_{s}-y_{d}$$

We set the sliding surface [29] as:

(36) $$s=c_{1}z_{1}+z_{2}+c_{0}\int_{0}^{t}\left(z_{s}-y_{d}\right)dt$$

where $c_{0}$ and $c_{1}$ are the coefficient of the sliding mode surface and the weight of each item in the controller.

When the system is in sliding mode motion, the following conditions are met:

(37) $$\dot{s}=c_{1}{\dot{z}}_{1}+{\dot{z}}_{2}+c_{0}e=c_{1}z_{2}+v+c_{0}e=0$$

Then,

(38) $$v_{eq}=-c_{1}z_{2}-c_{0}e$$

where $v_{eq}$ is called the equivalent control, and the equivalent control keeps the system state on the sliding mode surface.

We transform Equation (37) to obtain:

(39) $$\dot{s}=c_{1}{\dot{z}}_{1}+{\ddot{z}}_{1}+c_{0}e$$

After a Laplace transform, we can obtain:

(40) $$c_{1}Z_{1}\left(s\right)s+Z_{1}\left(s\right)s^{2}+c_{0}\left[Z_{1}\left(s\right)-Y_{d}\left(s\right)\right]=0$$

and then

(41) $$\frac{Z_{1}\left(s\right)}{Y_{d}\left(s\right)}=\frac{c_{0}}{s^{2}+c_{1}s+c_{0}}$$

The characteristic equation of the system is obtained as:

(42) $$s^{2}+c_{1}s+c_{0}=0$$

The pole assignment method is used to determine the values of $c_{0}$ and $c_{1}$. The assigned poles are $s_{1,2}=-40\pm j20$, and the expected characteristic polynomial is

(43) $$\left(s+40+j20\right)\left(s+40-j20\right)=s^{2}+80s+2000$$

Hence, $c_{0}=2000$, $c_{1}=80$.

Substituting the coefficient into Equation (38) we have

(44) $$v_{eq}=-80z_{2}-2000e$$

Due to the external disturbance of the system, the equivalent control cannot guarantee the robustness of the system, and therefore the switching control $v_{s}$ is introduced here. In order to realize the sliding mode motion, the system must meet the reachable condition $s\dot{s}\leq 0$. We construct the Lyapunov function $V=\frac{1}{2}s^{2}$. Since this function is semi-positive definite, the reachability condition can be satisfied by requiring its derivative $\dot{V}=s\dot{s}\leq 0$ [30]. Selecting the index approach rate gives

(45) $$\dot{s}=-\varepsilon \mathrm{sgn}\left(s\right)-\lambda s$$

where ε and λ are normal numbers. Symbolic functions are defined as [30]:

(46) $$\mathrm{sgn}\left(s\right)=\left\{\begin{array}{l} 1,s>0\\ 0,s=0\\ -1,s<0 \end{array}\right.$$

Then, substituting Equation (45) into $\dot{V}=s\dot{s}=-s\left[\varepsilon \mathrm{sgn}\left(s\right)+\lambda s\right]\leq 0$ shows that attainable conditions can be met.

According to the equality of Equations (37) and (45), we can obtain:

(47) $$v=-c_{1}z_{2}-c_{0}e-\varepsilon \mathrm{sgn}\left(s\right)-\lambda s=v_{eq}+v_{s}$$

where $v_{s}=-\varepsilon \mathrm{sgn}\left(s\right)-\lambda s$. Through continuous simulation and debugging, we can obtain ε=0.0001, λ=500.

**Figure 8:**
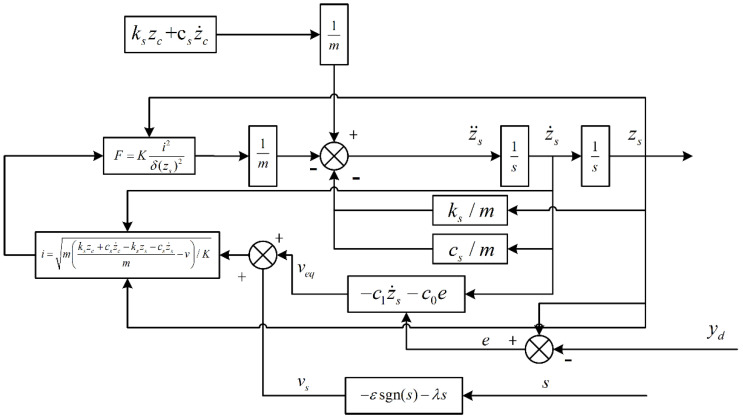
Structure block diagram of sliding mode control system.

The corresponding sliding mode control system was built according to the established equations, and its structure is shown in Figure 8.

## 4. Establishment of Simulation Test Model

### 4.1. Establishment of Pavement Spectrum Model

**Figure 9:**
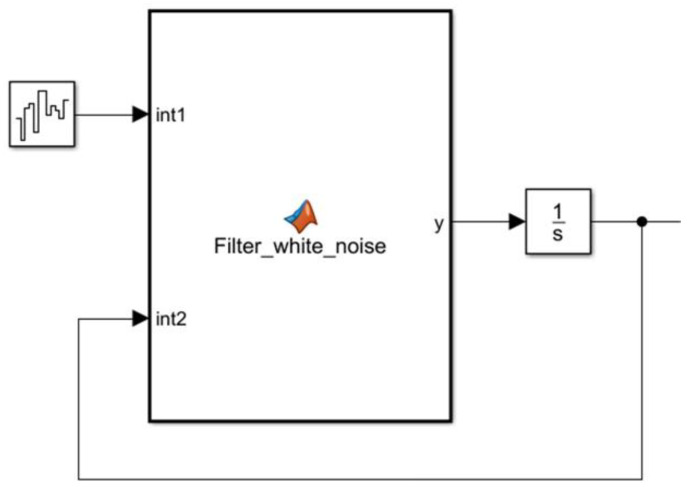
Time domain model of random pavement generation.

**Figure 10:**
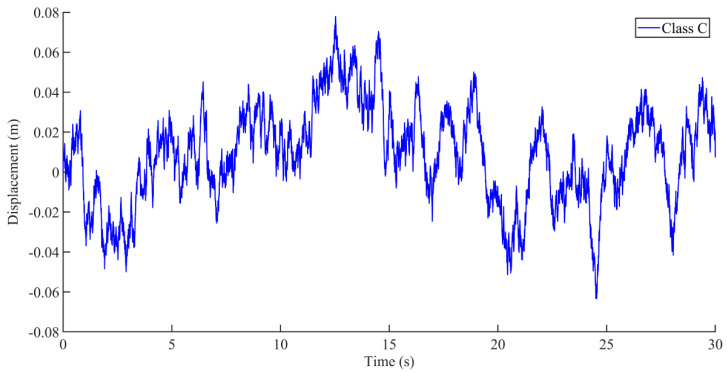
Road excitation under class C road surface (vehicle speed: 60 km/h).

A Simulink simulation model of road roughness based on filtered white noise was built, as shown in Figure 9. The pavement excitation (vehicle speed: 60 km/h) for a class C pavement was obtained according to the established pavement generation time-domain model, as shown in Figure 10.

### 4.2. Establishment of Simulation Model

**Figure 11:**
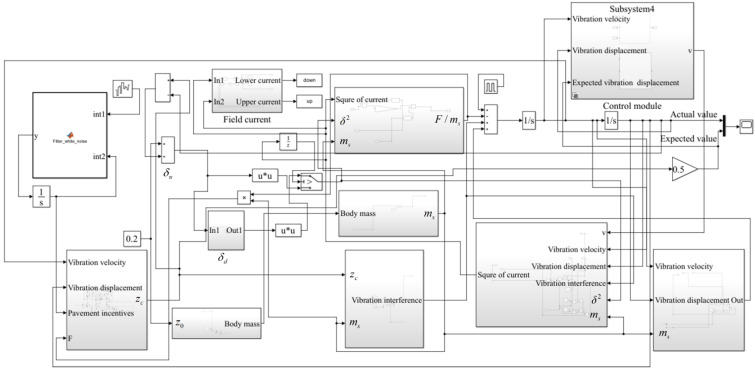
Simulink model of electromagnetic vibration damping system based on state feedback control and integral synovial control.

We set the distance between the electromagnets to 0.4 m, the seat mass to 10 kg, the weight of the person to 70 kg, the stiffness of the seat suspension to 35,000 N/m, and the initial position of the seat armature to 0.22 m away from the vehicle body. When the person sits on the seat, the armature position of the seat is 0.2 m. The control objective was to control the seat so that its vibration displacement was 50% of the vehicle body vibration displacement. The input is the road excitation when driving on a class C road at a speed of 60 km/h, and the electromagnetic coefficient K of the electromagnet was set at 12. The simulation was carried out with a fixed step length of 0.001 s, and the simulation time was 30 s. We set the external input interference acting on the seat as 800 N in amplitude, 5 s per cycle, and 15% in pulse width. The Simulink model of the electromagnetic vibration reduction system built based on state feedback control and integral sliding mode control is shown in Figure 11, and the system simulation parameter settings are shown in Table 1.

## 5. Simulation Results and Discussion

### 5.1. Vibration Study

**Figure 12:**
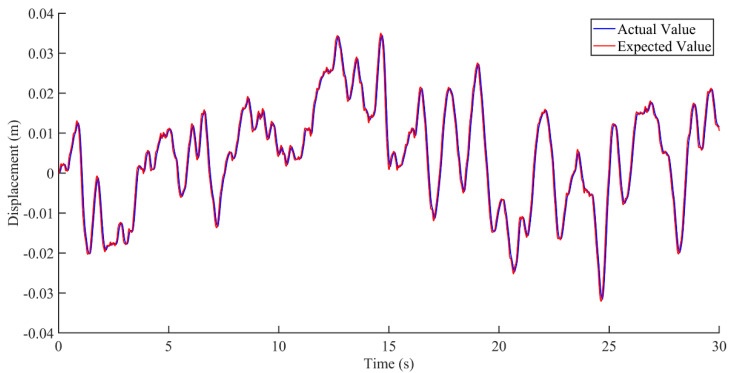
Comparison between actual value and expected value of vibration displacement based on state feedback control under the condition of no interference.

**Figure 13:**
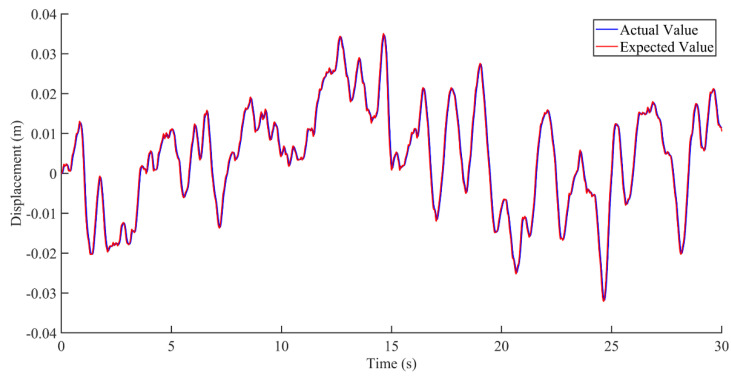
Comparison between actual value and expected value of vibration displacement based on integral sliding mode control under the condition of no interference.

**Figure 14:**
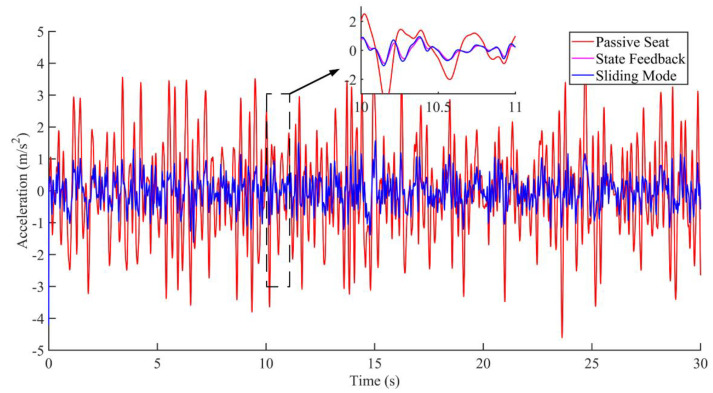
Acceleration comparison under the condition of no interference.

**Figure 15:**
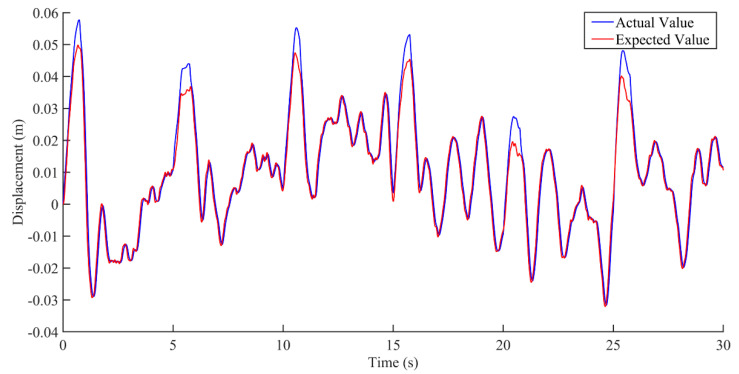
Comparison of actual and expected vibration displacement based on state feedback control under the condition of interference.

**Figure 16:**
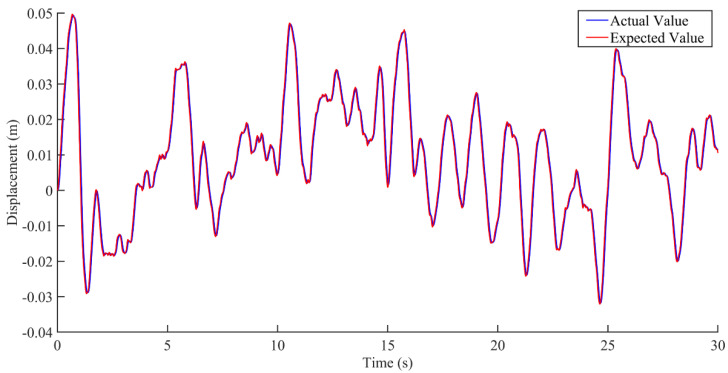
Comparison between the actual value and the expected value of vibration displacement based on the integral sliding mode control under the condition of interference.

**Figure 17:**
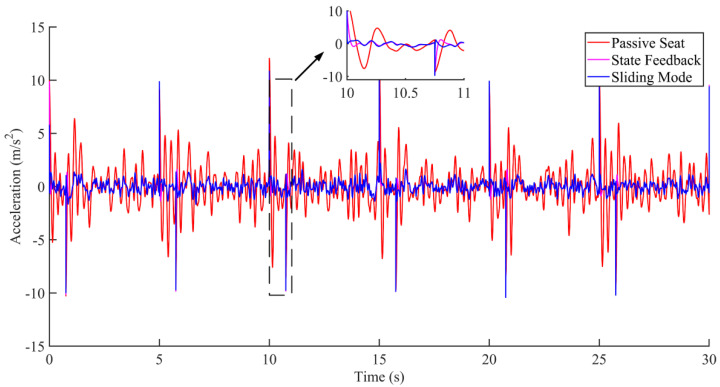
Acceleration comparison under the condition of interference.

Research on seat vibration reduction is mostly carried out on the vertical displacement and acceleration of the seat [21,22,23]. This paper mainly focuses on the displacement and acceleration of the active seat suspension and discusses the two cases of no interference and interference, as shown in Figure 12, Figure 13, Figure 14, Figure 15, Figure 16 and Figure 17. Combined with the analysis of the no-disturbance data in Figure 12 and Figure 13, it can be seen that in the case of no interference, the two control algorithms have a good control effect on the displacement. Based on the state feedback control, the average error between the actual value and the expected value was −1.85848 × 10^−5^ m, and the variance based on the mean was 4.59371 × 10^−6^ m^2^. Based on the integral sliding mode control, the average error between the actual value and the expected value was −1.5 × 10^−5^ m, and the variance based on the mean was 3.04127 × 10^−6^ m^2^. Combined with the analysis of the no-disturbance data in Figure 14, the RMS value of the passive seat acceleration was 1.438585 m/s^2^. The acceleration root mean square values of the integral sliding mode control and state feedback control were 0.478052 m/s^2^ and 0.426445 m/s^2^, respectively, and the vibration reduction effect was increased by 66.8% and 70.4%, respectively. In the case of no interference, the sliding mode control and the state feedback control had a good control effect on the acceleration.

According to the analysis of the interference data in Figure 15 and Figure 16, the control of the seat vibration displacement via integral sliding mode control was significantly better than via state feedback control in the presence of interference forces. It can be seen from the results in Figure 17 that the integral sliding mode control had a shorter response time and smaller overshoot in response to external interference than the state feedback control.

In summary, considering the anti-interference effect and robustness of the integral sliding mode control, the integral sliding mode control was preliminarily selected as the control method of the controller.

### 5.2. Calculation of Electromagnet Energy Consumption

**Figure 18:**
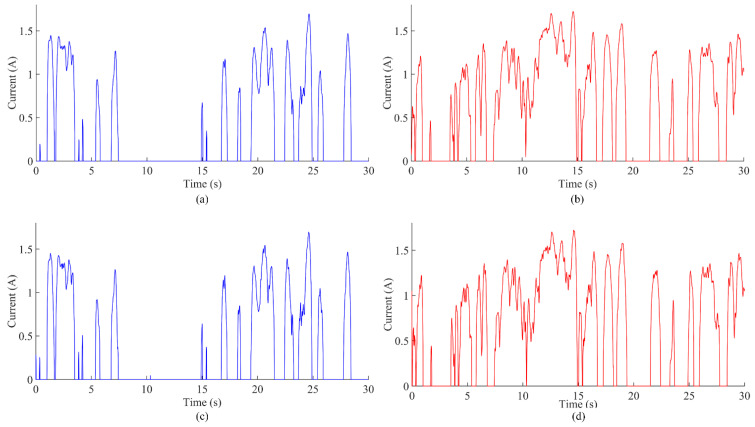
(**a**) Current value of the upper electromagnet under the state feedback control without interference; (**b**) current value of the lower electromagnet under the state feedback control without interference; (**c**) current value of the upper electromagnet under the integral sliding mode control without interference; (**d**) current value of the lower electromagnet under the integral sliding mode control without interference.

The electromagnet current values under two control states are shown in Figure 18. Figure 18a,b, respectively, show the current values of the upper and lower electromagnets under state feedback control without interference; Figure 18c,d, respectively, show the current values of the upper and lower electromagnets based on the integral sliding mode control without interference.

The electromagnet coefficient of the electromagnetic vibration damping system designed in this study was 12. According to Equation (7),

(48) $$S=\frac{12}{\pi \times 10^{-7}N^{2}}$$

If *N* = 10,000 turns, then *S* = 0.38 m^2^. The vehicle battery voltage was 48 V, and the maximum current that the battery can pass was $i_{\max}=5A$. The energy consumption of the active seat suspension is calculated on the premise that the maximum current is not exceeded.

The power consumption of the electromagnet per hour is calculated as follows.

For the non-interference state feedback control combined with the current value in Figure 18 and the vehicle battery voltage, it can be estimated that the energy consumption per hour is 174,000 j, i.e., 0.048 kw·h, which fully meets the current energy consumption requirements. For the non-interference sliding mode control combined with the current value in Figure 18 and the vehicle battery voltage, it can be estimated that the energy consumption per hour is 176,640 j, i.e., 0.049 kw·h, which also fully meets the current energy consumption requirements.

In summary, the energy consumption for state feedback control and integral sliding mode control is consistent, and both are maintained at a low level.

## 6. Conclusions

(1) In this paper, we took the vehicle seat as the research object and proposed an electromagnetic levitation active vibration reduction seat based on electromagnetic force control. The vibration attenuation of the seat was achieved by establishing a three-degrees-of-freedom dynamic “seat–body–tire” model, introducing electromagnetic force and applying methods such as integral sliding mode control. The main contribution of this paper is the new active seat suspension structure, which represents a structural innovation. The feasibility of this structure was proved via simulation.

(2) Comparing the acceleration and displacement of the electromagnetic levitation active seat suspension without interference and with interference, the results showed that the two methods of state feedback and integral synovial control had positive effects on the vehicle seat vibration reduction. However, the electromagnetic levitation active seat suspension based on integral sliding mode control had better anti-interference performance.

(3) The simulation was carried out on the condition that the vehicle was running on a level C road at a speed of 60 km/h. The results showed that the energy consumption of the electromagnetic levitation active seat suspension was low, and it had a good vibration suppression ability.

(4) This study is not limited to the application of an active seat suspension. The method could also be applied to the vibration isolation of ambulance stretcher beds, the vibration isolation of truck compartments, and the vibration isolation of on-board high-precision sensors. The control method could also be optimized further.

## Figures and Tables

**Table 1:** Simulation parameters of seat suspension.

Parameters	Physical Meaning	Value	Units
$m_{t}$	Tire mass	45	kg
$m_{c}$	Body mass	330	kg
$m_{s}$	Seat and human mass	80	kg
$k_{t}$	Tire stiffness	170,000	N/m
$k_{c}$	Body suspension stiffness	13,000	N/m
$k_{s}$	Seat suspension stiffness	35,000	N/m
$c_{s}$	Seat suspension damping	300	N/(m/s)
$c_{c}$	Body suspension damping	2000	N/(m/s)

## Data Availability

Not applicable.
